# The association between handgrip strength and depression in cancer survivors: a cross-sectional study

**DOI:** 10.1186/s12877-022-02795-0

**Published:** 2022-02-10

**Authors:** Xiao-Ming Zhang, Zhi-Biao Zhang, Wei Chen, Xinjuan Wu

**Affiliations:** 1 Department of Nursing, Chinese Academy of Medical Sciences - Peking Union Medical College, Peking Union Medical College Hospital (Dongdan Campus), Beijing, 100730 China; 2 Department of Breast Surgery, Dongguan Tungwah Hospital, Dongguan, 523110 China; 3 Beijing Key Laboratory of the Innovative Development of Functional Staple and the Nutritional Intervention for Chronic Disease, Building 6, No. 24 Courtyard, Jiuxianqiao Middle Road, Chaoyang District, Beijing, 100015 China; 4 Department of Clinical Nutrition, Department of Health Medicine, Chinese Academy of Medical Sciences - Peking Union Medical College, Peking Union Medical College Hospital (Dongdan Campus), No.1 Shuaifuyuan Wangfujing, Dongcheng District, Beijing, 100730 China

**Keywords:** Handgrip strength, Depression, Cancer survivors

## Abstract

**Background:**

The association between handgrip strength and depression in cancer survivors has been unexplored until now. We aim to examine the association between handgrip strength and depression in cancer survivors by using publicly available data (National Health and Nutrition Examination Survey).

**Methods:**

Two waves of data from the National Health and Nutrition Examination Survey, from 2011–2012 and 2013–2014, were extracted and combined to explore this important issue. We extracted maximum patient handgrip strength value (from both hands). The Patient Health Questionnaire (PHQ-9) was used to evaluate depression with a cut-off >  = 10 points indicating that patients had depressive symptoms. Other basic characteristics and health-related variables were also collected. We used Least Absolute Shrinkage and Selection Operator (LASSO) regression to select potential confounding factors. Multivariable linear or logistic regression models were adopted to explore whether handgrip strength as a continuous variable, or low handgrip strength, was associated with depressive symptoms.

**Results:**

There were 876 cancer survivors in our present total sample, with 403 (46.0%) males and 473 females (54.0%). The mean (SD) age of the entire group was 64.67 (13.81) years. The prevalence of depression and low handgrip strength was 12.90% and 16.7%, respectively. The results showed that handgrip strength was negatively associated with depressive symptoms in cancer survivors (OR = 0.95, 95%CI:0.92–0.99; *P* = 0.024). In addition, after adjusting for age, gender, race; marital status, polypharmacy, sleep disorder, arthritis, congestive heart failure, history of stroke, type of cancer, chronic coronary bronchitis and being overweight, cancer survivors with low handgrip strength had a 2.02-fold risk of depression, compared to those with normal handgrip strength (OR = 2.02,95%CI:1.07–3.81; *P* = 0.028).

**Conclusions:**

Our present study suggests that low handgrip strength, as a simple and modifiable parameter, is associated with a higher risk of depression in cancer survivors. Therefore, future larger-scale prospective cohort studies are warranted to determine this association.

**Supplementary Information:**

The online version contains supplementary material available at 10.1186/s12877-022-02795-0.

## Introduction

The number of cancer survivors is increasing, thanks to multiple advances in early-detection technologies and improved treatments for cancer [1]. Meanwhile, cancer survivors often suffer from depression symptoms, with an estimated figure of 20%, compared to 5% in the general population [2]. A previous meta-analysis has reported that the prevalence of major depression and minor depression was 15% and 20%, respectively [3]. Cancer patients who experience depression could be at increased risk of adverse outcomes, such as poor adherence to medical treatment [4], lower survival time [5], and increased likelihood of suicide [6], leading to the need for a tremendous amount of attention from clinicians and society alike. Therefore, early identification of risk and corresponding management of depression in cancer patients is essential.

Several factors—serious illness, female gender, social deprivation, and other health-related factors—are associated with depression [7]. Apart from these factors, recent studies have focused on assessing the relationship between functional limitations, such as low handgrip strength and depression [8]. Handgrip strength, as an indicator of muscle mass function, can easily be assessed using a dynamometer and common use to record muscular strength, and widely applied in different settings [9]. Handgrip strength has been linked to a higher risk of cardiovascular disease, metabolic syndrome, neurologic disease, and future disability [10]. A previous study indicated that muscle mass could influence depression by secreting myokines [11], including irisin and FGF21, which are supposed to mediate depression. Furthermore, muscle tissue is considered one of the largest exercise and endocrine metabolism organs, which was explored by a study on depression [12]. Patients with a decline in muscle strength might be unlikely to participate in physical activity, thus increasing their risk of depression [13].

The relationship between handgrip strength and depression among community-dwelling older adults has been well-explored. Previous studies have suggested that older adults with high handgrip strength had a lower risk of depressive symptoms [14, 15]. Furthermore, in a recent meta-analysis, handgrip strength was correlated with depression (Pooling OR = 0.85, 95% CI:0.80, 0.89), indicating handgrip strength as a protective factor for depression [16]. However, the majority of the studies included for meta-analysis were conducted in community or nursing home settings where the participants were relatively healthy. In addition, no research has been done on the relationship between handgrip strength and depression in cancer patients.

To our knowledge, no previous study has explored the association between handgrip strength and depression in cancer patients. Given the rapid increase in the number of cancer patients and a higher prevalence of depression among cancer patients, exploring whether handgrip strength is correlated to depression could help clinicians and nurses screen for this factor early on, and perform effective interventions to improve handgrip strength and alleviate depression. Eventually, this could possibly improve cancer survivors' quality of life. Therefore, the present study's aim was to investigate the association between handgrip strength and depression in cancer patients by using the public Internet database of the National Health and Nutrition Examination Survey. We hypothesized that, in cancer survivors, there is a negative relationship between handgrip strength and depression.

## Methods

### Study design and participants

This cross-sectional study of the National Health and Nutrition Examination Survey (NHANES) was to explore the overall picture of nutrition, health, and risk factors among residents in various states across the U.S. (Centers for Disease Control and Prevention-http://www.cdc.gov/nchs/nhanes.htm). This survey is based on the national-scale adoption of multistage and clustered sample methods, with 5,000 participants each year. Voluntary participants were asked to complete a physical examination. This study was approved by the Health Statistics Research Ethics Review Board from a national center. All individuals signed a written consent form.

In the present study, data including handgrip strength, depression, sleep disorder, cancer type, baseline characteristics, and other health-related variables were extracted from NHANES from 2011 to 2012 and from 2013 to 2014, aggregating for final analysis. There are 547 cancer survivors in the 2013–2014 data documentation and 488 cancer survivors in the 2011–2012 data documentation, resulting in 1,035 participants. We removed the participants who did not finish the handgrip strength and depression evaluation, leading to 876 participants in our final analysis.

### Cancer patients

Cancer patients were confirmed by asking the question (Have you ever been told by a doctor that you have a diagnosis of any type of cancer?). We selected the patients whose answer was "YES." Then, if the answer was YES, they were asked what kind of cancer it was.

### Handgrip strength

The muscle strength measurement details are depicted in the NHANES Procedure Manual. Briefly, those participants who were able to finish the test followed the standard procedure. Investigators explained the detailed information and asked participants to try their best to squeeze the hand-held dynamometer three times, recording the maximum value as the participant's final handgrip strength. In this present study, the definition of low handgrip strength was < 30 kg for males and < 20 kg for females [17].

### Depressive symptoms

According to the Questionnaire Instruments, participant depressive symptoms were measured by the Patient Health Questionnaire (PHQ-9), consisting of nine items, with each item ranging from 0 to 3 points [18]. The total PHQ-9 points ranged from 0 to 27. We categorized participants into those having depression and those without depression, with the cut-off being 10 points based on the previous study. This was reported as having good sensitivity (88%) and specificity (88%) for identifying major depression [19].

### Covariates definition

We extracted demographic characteristics, consisting of age, gender, education, race, marital status, and smoking. Of these factors, race was defined as non-Hispanic Black, non-Hispanic White, and others; marital status was classified as married, widowed or divorced, and other. Regarding education, the four categories included less than twelfth-grade education, high school, some college, and college graduate or above, and were confirmed. Other covariates, such as BMI, cancer diagnosis, sleep disorder, history of stroke, history of arthritis, history of being overweight, history of thyroid issues, history of chronic bronchitis, history of chronic coronary heart disease, history of gout and history of congestive heart failure (CHF), were also extracted. In addition, leisure-time physical activity was evaluated by the Global Physical Activity Questionnaire [20]. The detailed calculation method was reported in the previous study [21]. In brief, the minutes per week of leisure-time physical exercise were calculated, counted as frequency multiplied by duration of physical activity in one week. Individuals reported the frequency and duration of vigorous and moderate-intensity physical activity. We recorded the total minutes of physical activity for each intensity level, where vigorous intensities needed to double when we used moderate intensities as a reference. Finally, the metabolic equivalent of task value for each week was used as the formula: total minutes = (frequency * vigorous-intensity * 2 + frequency *moderate intensities). We combined vigorous and moderate intensities of physical activity each week. According to the guidelines on physical activity for cancer survivors, the classification of physical activity for cancer survivors is divided into three categories: zero min/week MVPA for inactive, < 150 min/week MVPA for insufficiently active, and ≥ 150 min/week MVPA indicating sufficient [22].

### Statistical analysis

Continuous variables, including age, BMI, and depression score, were present as means and SD, and categorical variables, including cancer type, gender, race, education, marital status, sleep disorder, and other health-related variables were displayed as frequency (%). For appropriateness, Student's test and chi-squared test or Fisher's Exact Test and Mann–Whitney tests were used to make these comparisons (poor handgrip strength versus normal handgrip strength; depression versus non-depression). In addition, generalized additive model (GAM) analysis was used to detect whether there is a non-linear relationship between handgrip strength and depression [23]. Before performing multivariable logistic regression analysis, we used Least Absolute Shrinkage and Selection Operator regression for variable selection. The results indicated that these variables (age group: >  = 65 years versus < 65 years, gender, race, marital status, polypharmacy, sleep disorder, arthritis, congestive heart failure, history of stroke, chronic coronary bronchitis, overweight, and cancer type) were selected. Finally, multivariable logistic regression analysis was adopted to determine the independent relationship between handgrip strength and depression after adjusting for potential confounding factors, including age group: >  = 65 years versus < 65 years; gender; race; marital status, polypharmacy, sleep disorder, arthritis, congestive heart failure, history of stroke, chronic coronary bronchitis, overweight, and cancer type. We also categorized handgrip strength into low handgrip strength and normal handgrip strength, according to the sarcopenia of the European consensus on definition and diagnosis, with cut-off values of (< 20 kg for females and < 30 kg for males). The relationship between low handgrip strength and depression was also detected by multivariable logistic regression analysis with adjustment of the same variables. In addition, multivariable linear regression analysis was used to detect the relationship between low handgrip strength and depression score after adjusting the same confounding factors. To identify these associations (low handgrip strength and depression), subgroup analyses were conducted in terms of variables (marital status, race, >  = 65 years versus 65 years, gender, sleep disorder, cogestive heart failure, polypharmacy, history of stroke, leisure-time physical activity, chronic bronchitis, and education). These variables, including arthritis, gout, congestive heart failure, chronic coronary heart disease, stroke, thyroid, and chronic bronchitis, had missing data; whereas the proportion of missing data was less than 5%. We have added the missing data as unrecorded data in corresponding variables in Tables 1 and 2. For categorical data, we have created a subgroup with the missing variable data. Finally, in the multivariable regression model, we entered the selected variable with all corresponding groups into the regression model. All statistics were conducted using software packages R and Empowerstats, with the significant P-value being < 0.05.

**Table 1:** Baseline Clinical Characteristics

Variables	Total sample	Normal handgrip strength	Low handgrip strength	Standardize diff	P-value
N	876	730	146		
Age (years)(mean, SD)	64.67 ± 13.81	62.78 ± 13.86	74.10 ± 8.75	0.98 (0.79, 1.16)	< 0.001
Depression score(mean, SD)	3.91 ± 5.50	3.73 ± 4.98	4.79 ± 7.56	0.17 (-0.01, 0.34)	0.033
BMI(mean, SD)	29.02 ± 6.57	29.23 ± 6.57	27.85 ± 6.43	0.21 (0.03, 0.40)	0.024
Handgrip strength(kg) (mean, SD)	32.53 ± 10.96	35.01 ± 10.02	20.15 ± 5.85	1.81 (1.61, 2.01)	< 0.001
Gender (n, %)				0.14 (-0.04, 0.31)	0.137
Male	403 (46.00%)	344 (85.36%)	59 (14.64%)		
Female	473 (54.00%)	386 (81.61)	87 (18.39%)		
Education (n, %)				0.38 (0.20, 0.55)	< 0.001
Less than 12 grades	161 (18.38%)	117(72.67%)	44 (27.33%)		
High school graduate	178 (20.32%)	146 (82.02%)	32 (17.98%)		
Some college	277 (31.62%)	239 (86.28%)	38 (13.72%)		
College graduate above	260 (29.68%)	228 (87.69%)	32 (12.31%)		
Race (n, %)				.12 (-0.06, 0.30)	0.458
Other	156 (17.81%)	130 (83.33%)	26 (16.67%)		
Non-Hispanic White	576 (65.75%)	475 (82.47%)	101 (17.53%)		
Non-Hispanic Black	144 (16.44%)	125 (86.81%)	19 (13.19%)		
Marital status (n, %)				0.45 (0.27, 0.63)	< 0.001
Married	493 (56.34%)	434 (88.03%)	59 (11.97%)		
Widowed or divorced	268 (30.63%)	198 (73.88%)	70 (26.12%)		
Other	114 (13.03%)	97 (85.09%)	17 (14.91%)		
Overweight (n, %)				0.08 (-0.10, 0.25)	0.404
Yes	345 (39.38%)	292 (84.64%)	53 (15.36%)		
No	531 (60.62%)	438 (82.49%)	93 (17.51%)		
Arthritis (n, %)				0.44 (0.26, 0.62)	< 0.001
Yes	455 (51.94%)	354 (77.80%)	101 (22.20%)		
No	417 (47.60%)	373 (89.45%)	44 (10.55%)		
Not recorded	4 (0.46%)	3 (75.00%)	1 (25.00%)		
Gout (n, %)				0.18 (0.00, 0.36)	0.115
Yes	81 (9.25%)	61 (75.31%)	20 (24.69%)		
No	794 (90.64%)	668 (84.13%)	126 (15.87%)		
Not recorded	1 (0.11%)	1 (100.00%)	0 (0.00%)		
CHF (n, %)				0.34 (0.17, 0.52)	< 0.001
Yes	59 (6.74%)	37 (62.71%)	22 (37.29%)		
No	815 (93.04%)	691 (84.79%)	124 (15.21%)		
Not recorded	2 (0.23%)	2 (100.00%)	0 (0.00%)		
CHD				0.31 (0.13, 0.49)	< 0.001
Yes	68 (7.76%)	45 (66.18%)	23 (33.82%)		
No	803 (91.67%)	681 (84.81%)	122 (15.19%)		
Not recorded	5 (0.57%)	4 (80.00%)	1 (20.00%)		
History of Stroke (n, %)				0.32 (0.14, 0.50)	< 0.001
Yes	74 (8.45%)	50 (67.57%)	24 (32.43%)		
No	800 (91.32%)	679 (84.88%)	121 (15.12%)		
Not recorded	2 (0.23%)	1 (50.00%)	1 (50.00%)		
Thyroid (n, %)				0.20 (0.02, 0.37)	0.022
Yes	178 (20.32%)	142 (79.78%)	36 (20.22%)		
No	695 (79.34%)	587 (84.46%)	108 (15.54%)		
Not recorded	3 (0.34%)	1 (33.33%)	2 (66.67%)		
Chronic bronchitis (n, %)				0.11 (-0.07, 0.29)	0.331
Yes	87 (9.93%)	70 (80.46%)	17 (19.54%)		
No	787 (89.84%)	659 (83.74%)	128 (16.26%)		
Not recorded	2 (0.23%)	1 (50.00%)	1 (50.00%)		
Liver disease (n, %)				0.12 (-0.06, 0.29)	0.172
Yes	61 (6.96%)	47 (77.05%)	14 (22.95%)		
No	815 (93.04%)	683 (83.80%)	132 (16.20%)		
Depression (n, %)				0.12 (-0.06, 0.30)	0.162
No	763 (87.10%)	641 (84.01%)	122 (15.99%)		
Yes	113 (12.90%)	(78.76%)	24 (21.24%)		
Sleep disorder (n, %)				0.14 (-0.04, 0.32)	0.121
Yes	338 (38.58%)	290 (85.80%)	48 (14.20%)		
No	538 (61.42%)	440 (81.78%)	98 (18.22%)		
Leisure time physical activity (n, %)				0.53 (0.35, 0.70)	< 0.001
Inactive	562 (64.16%)	444 (79.00%)	118 (21.00%)		
Insufficiently active	76 (8.68%)	63 (82.89%)	13 (17.11%)		
Sufficiently active	238 (27.17%)	223 (93.70%)	15 (6.30%)		
Polypharmacy (n, %)				0.38 (0.19, 0.56)	< 0.001
< 5	453 (57.93%)	394 (86.98%)	59 (13.02%)		
> = 5	329 (42.07%)	250 (75.99%)	79 (24.01%)		
Type of cancer (n, %)				0.29 (0.11, 0.47)	0.201
Others	176 (20.18%)	148 (84.09%)	28 (15.91%)		
Skin	202 (23.17%)	168 (83.17%)	34 (16.83%)		
Prostate	121 (13.88%)	101 (83.47%)	20 (16.53%)		
Melanoma	58 (6.65%)	53 (91.38%)	5 (8.62%)		
Lung	25 (2.87%)	19 (76.00%)	6 (24.00%)		
Gynecological	110 (12.61%)	97 (88.18%)	13 (11.82%)		
Colon	45 (5.16%)	36 (80.00%)	9 (20.00%)		
Breast	135 (15.48%)	104 (77.04%)	31 (22.96%)		

Not recorded: missing data; *CHD* Chronic coronary heart disease; *CHF* Congestive Heart Failure

**Table 2:** Comparison between depression and non-depression

Variables	Statistics	Depression	P-value
Age(mean, SD)	64.67 ± 13.81	0.97 (0.96, 0.99)	< 0.0001
Gender(n, %)			
Male	403 (46.00%)	1.0	
Female	473 (54.00%)	1.59 (1.05, 2.39)	0.0272
Overweight (n, %)			
Yes	345 (39.38%)	1.0	
No	531 (60.62%)	0.62 (0.42, 0.92)	0.0184
Arthritis (n, %)			
Yes	455 (51.94%)	1.0	
No	417 (47.60%)	0.55 (0.36, 0.83)	0.0046
Not recorded	4 (0.46%)	5.32 (0.74, 38.38)	0.0974
Gout(n, %)			
Yes	81 (9.25%)	1.0	
No	794 (90.64%)	1.06 (0.53, 2.12)	0.8727
Not recorded	1 (0.11%)	NA	
CHF(n, %)			
Yes	59 (6.74%)	1.0	
No	815 (93.04%)	0.61 (0.31, 1.21)	0.1594
Not recorded	2 (0.23%)	NA	
CHD(n, %)			
Yes	68 (7.76%)	1.0	
No	803 (91.67%)	0.74 (0.37, 1.45)	0.3783
Not recorded	5 (0.57%)	3.45 (0.52, 23.15)	0.2015
History of Stroke(n, %)			
Yes	74 (8.45%)	1.0	
No	800 (91.32%)	0.38 (0.22, 0.67)	0.0008
Not recorded	2 (0.23%)	2.89 (0.17, 48.59)	0.4601
Thyroid(n, %)			
Yes	178 (20.32%)	1.0	
No	695 (79.34%)	1.29 (0.77, 2.18)	0.3363
Not recorded	3 (0.34%)	4.18 (0.36, 48.35)	0.2516
Chronic bronchitis(n, %)			
Yes	87 (9.93%)	1.0	
No	787 (89.84%)	0.33 (0.20, 0.56)	< 0.0001
Not recorded	2 (0.23%)	NA	
Liver disease(n, %)			
Yes	61 (6.96%)	1.0	
No	815 (93.04%)	0.42 (0.23, 0.78)	0.0060
Sleep disorder (n, %)			
Yes	338 (38.58%)	1.0	
No	538 (61.42%)	0.25 (0.17, 0.39)	< 0.0001
BMI (mean, SD)	29.02 ± 6.57	1.04 (1.01, 1.07)	0.0162
Low handgrip strength (n, %)			
No	730 (83.33%)	1.0	
Yes	146 (16.67%)	1.42 (0.87, 2.31)	0.1639
Leisure time physical activity (n, %)			
Inactive	562 (64.16%)	1.0	
Insufficiently active	76 (8.68%)	0.64 (0.28, 1.44)	0.2807
Sufficiently active	238 (27.17%)	0.87 (0.55, 1.38)	0.5633
Race(n, %)			
Other	156 (17.81%)	1.0	
Non-Hispanic White	576 (65.75%)	0.62 (0.38, 1.03)	0.0633
Non-Hispanic Black	144 (16.44%)	1.21 (0.66, 2.20)	0.5338
Education (n, %)			
Less than 12 grades	161 (18.38%)	1.0	
High school graduate	178 (20.32%)	0.66 (0.37, 1.17)	0.1534
Some college	277 (31.62%)	0.68 (0.41, 1.14)	0.1404
College graduate above	260 (29.68%)	0.26 (0.14, 0.50)	< 0.0001
Marital status (n, %)			
Married	493 (56.34%)	1.0	
Widowed or divorced	268 (30.63%)	2.34 (1.50, 3.65)	0.0002
Other	114 (13.03%)	2.71 (1.56, 4.73)	0.0004
Polypharmacy (n, %)			
< 5	453 (57.93%)	1.0	
> = 5	329 (42.07%)	2.35 (1.53, 3.60)	< 0.0001
Handgrip strength(kg) (mean, SD)	32.53 ± 10.96	0.98 (0.96, 1.00)	0.0409
Type of cancer (n, %)			
Others	176 (20.18%)	1.0	
Skin	202 (23.17%)	0.81 (0.42, 1.55)	0.5270
Prostate	121 (13.88%)	0.81 (0.38, 1.72)	0.5878
Melanoma	58 (6.65%)	1.18 (0.49, 2.83)	0.7093
Lung	25 (2.87%)	0.31 (0.04, 2.39)	0.2599
Gynecological	110 (12.61%)	1.95 (1.02, 3.73)	0.0429
Colon	45 (5.16%)	1.36 (0.54, 3.43)	0.5155
Breast	135 (15.48%)	1.28 (0.66, 2.48)	0.4571

Not recorded: missing data; *CHD* Chronic coronary heart disease, *CHF* Congestive Heart Failure, *NA* not available

## Results

### Baseline Clinical Characteristics

There were 876 cancer cases in this study. This included 202 skin cancers (23.1%), 121 prostate cancers (13.88%), 58 melanoma cancers (6.65%), 25 lung cancers (2.87%),110 gynecological cancers (12.61%), 45 colon cancers (5.16%), 135 breast cancers (15.48%), and 176 other cancers (20.18%). The mean (SD) age of the entire group was 64.67 (13.81) years, with 403 (46.0%) males and 473 female(54.0%). The prevalence of depression and low handgrip strength was 12.90% and 16.7%, respectively. Of these patients, 329 (42.07%) participants experienced polypharmacy and 562 (64.16%) patients did not participate in any physical activity. Other information is presented in Table 1.

### Baseline characteristics between low handgrip strength and normal handgrip strength

Overall, cancer patients with low handgrip strength were prone to be older, have lower BMI, and more likely to be physically inactive. In addition, the proportion of polypharmacy and the depression score mean were higher in cancer patients with low handgrip strength, compared to those with normal handgrip strength. Patients with a history of stroke, chronic coronary heart disease, arthritis, thyroid or congestive heart failure tended to have a higher proportion of low handgrip strength. (Table 1).

### Univariate analysis of the factors related to depression

The results of univariate analysis showed that age, being female, being overweight, BMI, handgrip strength and having a sleep disorder, arthritis, history of stroke, chronic bronchitis, liver disease and polypharmacy were all factors that were associated with depression. In addition, unmarried cancer patients were more at risk for depression. Other related variables are displayed in Table 2.

### The relationship between handgrip strength or low handgrip strength and depression risk

Multivariable logistic or linear regression analysis was conducted to determine the relationship between handgrip strength or low handgrip strength and depression in cancer patients. The results showed that handgrip strength was a protective factor for depression after adjusting for age group and gender, the OR being 0.95(95%CI:0.92–0.98; *P* = 0.003). After fully adjusting for potential confounding factors, this association still existed (OR = 0.95,95%CI:0.92–0.99; *P* = 0.024). When handgrip strength was classified into either low handgrip strength or normal handgrip strength, the results also indicated that cancer patients with low handgrip strength had an increased risk of depressive symptoms(OR = 2.02,95%CI:1.07–3.81; *P* = 0.028), after adjusting for age (< = 65 years versus < 65 years), gender, race, marital status, polypharmacy, sleep disorder, arthritis, congestive heart failure, history of stroke, chronic coronary bronchitis, being overweight, and type of cancer (Table 3). In addition, we have provided the results of the relationship between low handgrip strength and PHQ-9 scores by multivariable linear regression, and the results showed a similar conclusion [β = 1.13 (95%CI:0.14, 2.12); *P* = 0.024] (Supplemental Table 1).

**Table 3:** Multivariable regression analysis of the association between handgrip strength and the risk of depression in different adjusted models

Exposure	Adjust I OR (95%CI)		Adjust II OR (95%CI)	P-value	Adjust III P-value / OR (95%CI)	P-value
Low handgrip strength						
No	Reference		Reference		Reference	
Yes	2.070 (1.208, 3.548)	0.008	1.904 (1.057, 3.427)	0.031	2.02(1.07, 3.81)	0.028
	Adjust I OR (95%CI) /		Adjust I OR (95%CI) /	P-value	Adjust II OR (95%CI)	P-value
Handgrip strength^a^(per l kg)	0.957 (0.929, 0.985)	0.003	0.957 (0.927, 0.989)	0.007	0.958 (0.925, 0.993)	0.024

^a^Handgrip strength as continuous variable

Results: OR (95%CI) P-value

Outcome: depression

Exposure: low handgrip strength or handgrip strength as continuous variable

Adjust I model adjusted for: Age; gender

Adjust II model adjusted for: age (< = 65 years versus < 65 years); gender; stroke; polypharmacy; arthritis

Adjust III model adjusted for: age (< = 65 years versus < 65 years); gender; race; marital status, polypharmacy, sleep disorder, arthritis, congestive heart failure, history of stroke, chronic coronary bronchitis; overweight; type of cancer

### Non-linear relationship analyses

**Fig. 1:**
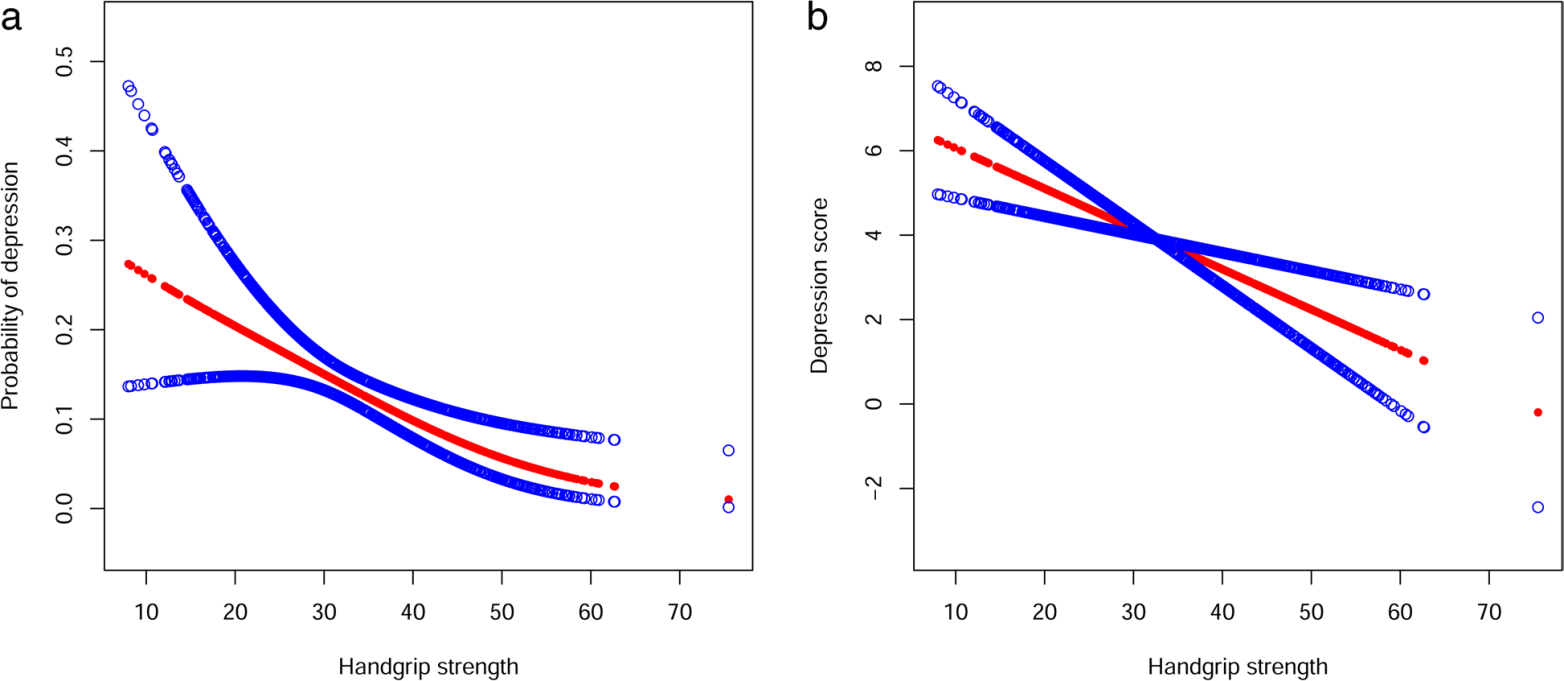
**a** A generalized additive model shows a linear association between handgrip strength and depression risk. **b** A linear relationship between handgrip strength and depression score by a generalized additive model

The generalized additive model (GAM) analysis was adopted to determine whether there is a non-linear relationship between handgrip strength and depression, and the results suggested handgrip strength was negatively associated with the risk of depression, meaning that the possibility of depression will decrease with increased handgrip strength. (Shown in Fig. 1a). In addition, with increased handgrip strength, depression scores also decreased (Fig. 1b).

### Subgroup analysis between low handgrip strength and depression in terms of different variables

**Fig. 2:**
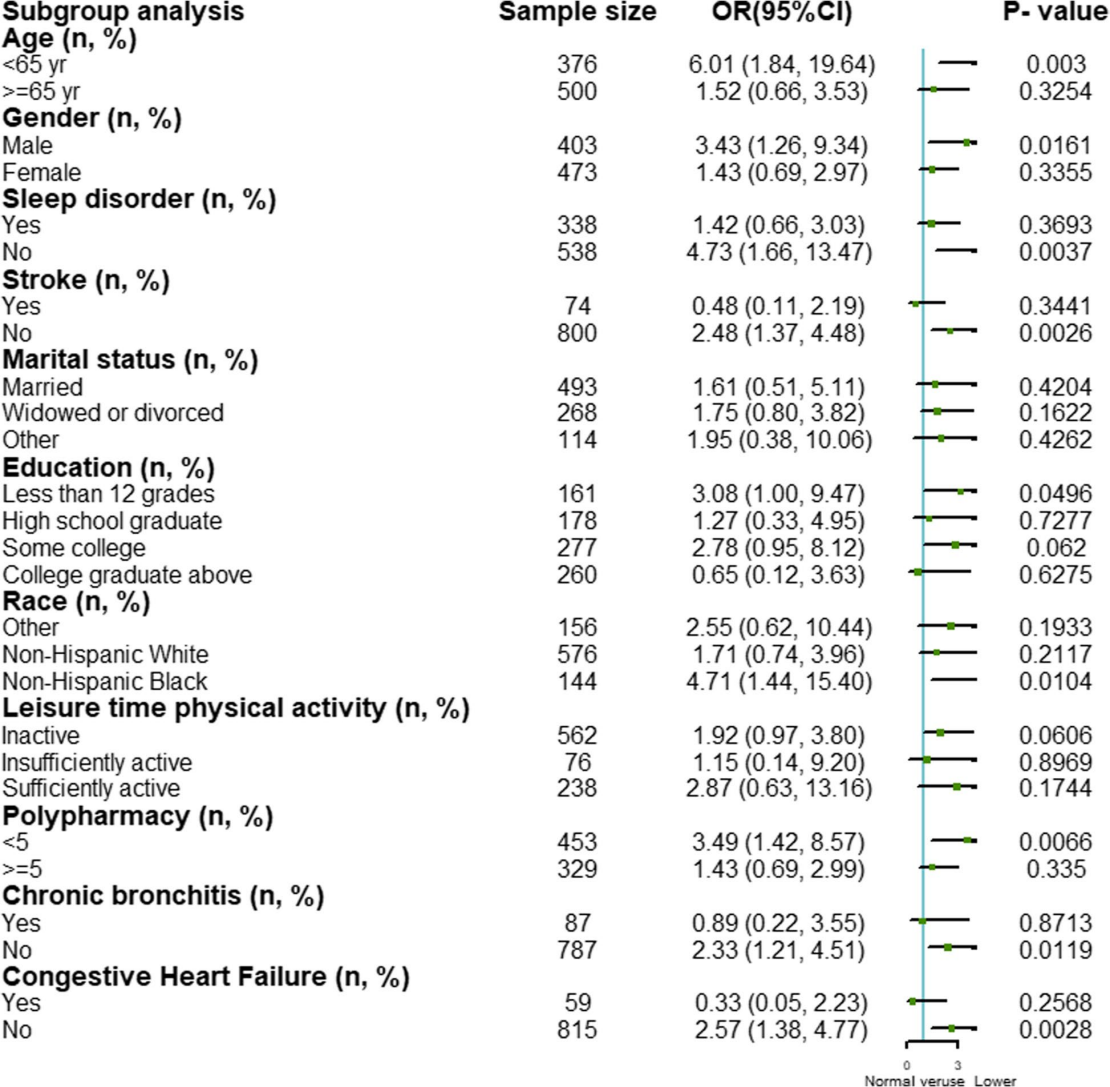
Subgroup analysis between low handgrip strength and depression in terms of different variables

The subgroup analysis showed that the association between low handgrip strength and depression in cancer patients was almost unchanged in various strata, indicating this was reliable and stable (Fig. 2).

## Discussion

This present study showed that cancer patients with low handgrip strength had a greater risk of depression than those with normal handgrip strength, after controlling for potential confounding factors. Our results support a hypothesis that handgrip strength is inversely correlated to depressive symptoms in cancer survivor.

This is the first study, to the best of the authors' knowledge, to investigate the relationship between handgrip strength and depression in cancer survivors. Many studies have examined the relationship between handgrip strength and depression in community-dwelling populations. In a cross-sectional study consisting of 24,109 Chinese adults aged 41.5 (SD = 11.9) years, the authors reported that people with stronger handgrip were found to be at lower risk of depression, and this association was particularly strong in females [24]. Furthermore, another prospective cohort study conducted among rural Chinese populations reported the reverse association between handgrip and depression [25]. Our study is in line with these abovementioned studies. However, most of these studies were conducted with relatively healthy people; only a few studies focused on hospitalized patients. A 2020 study reported this association in patients with chronic disease, with inconsistent results [26]. According to the study results, participants with a high strength tertile had decreased depression—both patients without disease or without metabolic disease; however, this association was not observed in patients with arthritis, and this needs more study. Our study focused on a special population, cancer survivors, who are at risk of depression [27]. Additionally, cancer patients with comorbid depression experienced an adverse impact in terms of treatment and recovery. Therefore, early prevention and treatment for depression is essential for cancer survivors.

Prior studies mainly focused on other depression risk factors, consisting of social factors (family, social support, stressful life event), cancer characteristics (type of cancer, recurrence, prognosis), cancer treatment (radiotherapy, chemotherapy, treatment burden), individual characteristics (age, gender, marital status) and psychological response to diagnosis. To the best of our knowledge, no prior study has explored handgrip strength, a modifiable parameter, in association with depression in cancer patients. Obviously, handgrip strength has many merits: it is simple, convenient, and not time-consuming, compared to these abovementioned factors, which are widely used in clinical and other primary community healthcare settings [28]. The most significant merit is that handgrip strength can be modified by intervention. Given that our study indicates that lower handgrip strength increases the risk of depression (OR = 2.02,95%CI:1.07–3.81; *P* = 0.028), it is reasonable to assume that improvements in handgrip strength by multiple measures, such as resistance training programs and nutritional interventions, can reduce the risk of depression, positively impacting cancer patient treatment and recovery. In a randomized controlled trial, the author reported that aerobic exercise intervention increased muscle mass in patients with major depression, which was beneficial in improving depression [29].

The mechanism underlying the link between poor handgrip strength and depression is complex. First, low handgrip strength as the core component for defining sarcopenia, which was reported by multiple studies [30, 31], was associated with a high risk of depression. Second, cancer patients with low handgrip strength are more likely to be physically inactive. In this present study, cancer survivors with low handgrip strength experienced a higher proportion of inactivity than those with normal handgrip strength (80.8% versus 60.8%). A recent meta-analysis covering 97 randomized controlled trials reported that exercise programs can reduce the risk of depressive symptoms, with the pooled SMD being − 0.276 ( 95% CI − 0.482 to − 0.070) [32]. Therefore, cancer patients with low handgrip strength, participating in little to no physical activity, would not gain the beneficial effects of reduced depression produced by exercise. Third, some studies reported that muscle–brain crosstalk can be mediated by myokines and metabolites, which are secreted by muscles, and play a role in regulating hippocampal function, which is closely related to depression [33]. Although there are some possible reasons to explain the link between low handgrip strength and depression, future studies are warranted to explore this underlying mechanism.

This study possesses both strengths and drawbacks. First, to our knowledge, this is the first study to examine the relationship between handgrip strength and depressive symptoms among cancer survivors, a fundamental issue for the prevention and management of depression in oncology patients. Second, our study suggests that low handgrip strength is significantly associated with depressive symptoms in cancer patients. Therefore, we need to pay more attention to handgrip strength in cancer survivors. Given the characteristics of modification of handgrip strength, appropriate and personalized exercises and nutritional programs might be beneficial to improve handgrip strength and alleviate depression in cancer survivors. Third, our study used comprehensive statistical analysis, such as Lasso regression, by using shrinkage, to better select variables to minimize multicollinearity. However, some drawbacks must also be mentioned. First, as this was a cross-sectional study, it limited our ability to identify a completely causal association. More prospective cohort studies for further exploration are required. Second, providing information about cancer treatment, such as what kind of treatment (surgery with chemotherapy and/or radiation therapy or immunotherapy), could provide more information. However, the original database did not contain these important variables. In the original database, cancer survivors were asked when the patient was first diagnosed with cancer. However, the item answer was not a specific number; therefore, it was difficult to calculate the length of cancer survivorship.

## Conclusion

Our study indicates that cancer survivors with low handgrip strength had about a 2.02-fold risk of depression, suggesting that improvements in handgrip strength might be beneficial for depressive symptoms among cancer survivors. The relationship between handgrip strength and depressive symptoms among cancer survivors should be investigated in a large cohort study, to clarify this finding.

## Supplementary Information

**Additional file 1: Supplemental Table1.** Multivariable linear regression analysis of the association between handgrip strength and the depression score in different adjusted models.

## Data Availability

The datasets generated and/or analyzed during the current study are available in the NHANES database.

## Abbreviations

LASSO: Least Absolute Shrinkage and Selection Operator
PHQ-9: Patient Health Questionnaire
NHANES: National Health and Nutrition Examination Survey
CHF: Congestive Heart Failure
CHD: Chronic coronary heart disease
