# Supplementary material for: The association between handgrip strength and depression in cancer survivors: a cross-sectional study
Source: BMC Geriatr. 2022 Feb 10;22:111. doi: 10.1186/s12877-022-02795-0 (PMC8829989; doi:10.1186/s12877-022-02795-0)
Supplement: Supplementary file 1 — Additional file 1: Supplemental Table1. Multivariable linear regression analysis of the association between handgrip strength and the depression score in different adjusted models. [file 12877_2022_2795_MOESM1_ESM.doc]

Supplemental Table1 Multivariable regression analysis of the association between handgrip strength and the depression score in different adjusted models

| Exposure | Non-adjusted  β (95%CI) P-value / | Adjust I  β (95%CI) P-value / | Adjust II  β (95%CI) P-value / |
| --- | --- | --- | --- |
|  |  |  |  |
| Low handgrip strength |  |  |  |
| No | Reference | Reference | Reference |
| Yes | 1.63 (0.63, 2.63) 0.001 | 1.02 (0.02, 2.03) 0.045 | 1.13 (0.14, 2.12) 0.024 |

Outcome: depression score

Exposure: low handgrip strength

Adjust I model adjusted for: Age; gender

Adjust II model adjusted for: age (<=65 years versus <65 years); gender; stroke; polypharmacy; arthritis

Adjust III model adjusted for: age (<=65 years versus <65 years); gender; race; marital status, polypharmacy, sleep disorder, arthritis, congestive heart failure, history of stroke, chronic coronary bronchitis; overweight; type of cancer.
